# Detection of *Wolbachia* Infections in Natural and Laboratory Populations of the Moroccan Hessian Fly, *Mayetiola destructor* (Say)

**DOI:** 10.3390/insects11060340

**Published:** 2020-06-02

**Authors:** Naima Bel Mokhtar, Amal Maurady, Mohammed Reda Britel, Mustapha El Bouhssini, Costas Batargias, Panagiota Stathopoulou, Elias Asimakis, George Tsiamis

**Affiliations:** 1 Laboratory of Innovative Technologies, National School of Applied Sciences of Tangier, Abdelmalek Essaâdi University, BP: 1818 Tanger, Morocco; naima1503@gmail.com (N.B.M.); amal.maurady.ma@gmail.com (A.M.); mrbritel@hotmail.com (M.R.B.); 2 International Center for Agricultural Research in the Dry Areas (ICARDA), P.O. Box 6299 Rabat, Morocco; M.Bohssini@cgiar.org; 3 Department of Animal Production, Fisheries and Aquaculture, University of Patras, Nea Ktiria, 30200 Messologhi, Greece; cbatargias@upatras.gr; 4 Department of Environmental Engineering, University of Patras, 2 Seferi St, 30100 Agrinio, Greece; panayotastathopoulou@gmail.com (P.S.); eliasasim@gmail.com (E.A.)

**Keywords:** biological control, Hessian fly, high throughput sequencing, HTS, 16S rRNA gene

## Abstract

*Mayetiola destructor* (Hessian fly) is a destructive pest of wheat in several parts of the world. Here, we investigated the presence of reproductive symbionts and the effect of the geographical location on the bacterial community associated to adult Hessian flies derived from four major wheat producing areas in Morocco. Using specific 16S rDNA PCR assay, *Wolbachia* infection was observed in 3% of the natural populations and 10% of the laboratory population. High throughput sequencing of V3-V4 region of the bacterial 16S rRNA gene revealed that the microbiota of adult Hessian flies was significantly influenced by their native regions. A total of 6 phyla, 10 classes and 79 genera were obtained from all the samples. Confirming the screening results, *Wolbachia* was identified as well in the natural Hessian flies. Phylogenetic analysis using the sequences obtained in this study indicated that there is one *Wolbachia* strain belonging to supergroup A. To our knowledge, this is the first report of *Wolbachia* in Hessian fly populations. The observed low abundance of *Wolbachia* most likely does not indicate induction of reproductive incompatibility. Yet, this infection may give a new insight into the use of *Wolbachia* for the fight against Hessian fly populations.

## 1. Introduction

Hessian fly, *Mayetiola destructor* (Say) (Diptera: Cecidomyiidae), is one of the most destructive insect pests of wheat in North Africa, North America and southern Europe [1]. In Morocco, damage caused by the Hessian fly can result in total crop loss if fall infestations are high and coincide with young stages of the crop [2]. The life cycle of the Hessian fly consists of eggs, three larval instars, pupae and adults. Adult flies resemble to mosquitos and have a short life span of 2 days for males and from 2 to 5 days for females with reduced flying capability [3,4,5]. Adults do not feed but mate and lay eggs on the upper surfaces of young wheat leaves. After hatching, first instar larvae crawl to the base of seedlings, where they establish a feeding site. The Hessian fly larvae and pupae develop in the same position and feed only during the first and second larval instars [6]. Feeding on the base of the seedlings causes stunting of the infested plants, which eventually become dark green and stop growing. This type of damage is observed mainly on young plants, while plants attacked at a more developed stage are not completely killed, but undergo a shortening of the internodes, and their yields in grain and straw are reduced [6,7,8]. Currently, several control methods, such as the use of classical chemical control and resistant cultivars are applied for managing Hessian fly infestations [2,9,10,11]. With the high demand of environmentally friendly alternatives, we address hereby, a biological and innovative pest management approach based on microorganisms associated with the insects. It is known that a diverse array of bacterial species is widespread in insects and has a tremendous variety of impacts which engage in obligatory or facultative symbioses, ranging from parasitism to mutualism. Bacterial symbionts have been reported to affect host biology on aspects of development, nutrition, reproduction, and fitness [12,13,14,15,16,17,18].

Recently, reproductive endosymbionts have attracted attention for their potential as new biocontrol agents, with the most studied symbionts in this area belonging to *Wolbachia* [19,20,21]. *Wolbachia* are obligatory intracellular and maternally inherited bacteria infecting a number of invertebrates especially insects [19]. *Wolbachia* affect the reproduction of their host by several strategies allowing them to persist and spread rapidly in host population. Several studies show that *Wolbachia* are able to induce thelytokous parthenogenesis, male killing, feminization of genetic males and cytoplasmic incompatibility (CI) which is the most frequent and best studied effect that *Wolbachia* have on their hosts [22,23]. *Wolbachia* was also reported to provide fitness benefit to their hosts by influencing fecundity, nutrition, development and providing resistance to pathogens [24,25,26,27,28,29,30,31,32].

Apart from *Wolbachia*, other inherited symbionts that can alter the biology of their insect hosts exist. Among them, *Spiroplasma* are mainly extracellular bacteria belonging to the phylum Tenericutes, known to cause selective death of male offspring in *Drosophila* flies and in a nymphalid butterfly [33,34]. Furthermore, *Spiroplasma* has been found to protect the infected host against natural enemies [34,35,36]. The endosymbiont *Cardinium* belonging to the Bacteroidetes phylum, also causes various reproductive phenotypes in numerous hosts, including cytoplasmic incompatibility, feminization and parthenogenesis [37,38,39,40,41]. *Arsenophonus*, belonging to the phylum Proteobacteria, establish diverse symbiotic interactions with approximately 5% of insect species, and mainly induce male killing phenomena [42,43].

To date, there are very few studies on the characterization of the bacterial community associated to Hessian fly populations. Bansal et al. 2011 based on classical sequencing, showed that for both culture-dependent and -independent methods, laboratory Hessian fly adults harbor four phyla: Proteobacteria, Firmicutes, Actinobacteria, and Bacteroidetes with difference in proportion between both methods [44]. Using culture-dependent methods, Hessian fly adults were mainly dominated by *Bacillus* genus (62.5%). Very low levels of proteobacterial genera were detected (less than 3.1%). However, using culture-independent method Hessian fly adults were dominated mainly by members of Proteobacteria with *Ochrobactrum* as the most abundant genus (55%). These differences were explained by the inability of some bacterial genera belonging to Proteobacteria phylum to grow on laboratory growth media. In regards to the *Wolbachia*-infection, Johnson et al. 2004 [45] tested by Long PCR technique the presence of *Wolbachia* in Hessian fly adults from 30 different geographic origins within the North America, the Mediterranean basin, Southwest Asia, and New Zealand obtained between 2000 and 2003 in addition to six laboratory populations. The results were negative for all the natural and laboratory populations.

In the present study, we aimed to screen laboratory and natural populations of Hessian fly for reproductive symbionts. The screening results were positive only for *Wolbachia* infections. The characterization of these *Wolbachia* strains was based on the use of 16S rRNA gene. In addition, we report on the bacterial symbionts associated to natural populations of adult Hessian flies using a high throughput sequencing (HTS) approach based on the Illumina-MiSeq platform.

## 2. Materials and Methods

### 2.1. Sample Collection and DNA Isolation

Four different Hessian fly populations were obtained from bread wheat from the major wheat producing areas in Morocco: Doukkala, Fes, Chaouia, and Safi (Table 1). The plants containing Hessian fly pupae were collected in winter 2017 and 2018 and they were reared in a growth room under constant conditions (Temperature 25 ± 1 °C, Relative Humidity 51.7%) from which adults were allowed to emerge. The first generation of adults were collected. A laboratory population kept at the International Center of Agricultural Research in Dry Areas (ICARDA) in Rabat, Morocco, was also included in the screening analysis (Table 1). All samples collected were placed separately (one adult per tube) in 100% ethanol and stored at room temperature until use. Before the DNA extraction, samples were rinsed with sterile water then the DNA of the whole fly was isolated using a modified CTAB (Cetyl Trimethyl Ammonium Bromide) method [46]. The quality and quantity of DNA samples was tested using a Q5000 micro-volume UV Vis spectrophotometer (Quawell Technology, San Jose, CA, USA). DNA samples were stored in Eppendorf tubes at −20 °C until PCR amplification and amplicon sequencing analysis.

### 2.2. Screening of Reproductive Symbionts and Sanger Sequencing

In total, 164 samples from wild populations and 80 samples from the laboratory colony were assayed for the presence of *Wolbachia*, *Spiroplasma*, *Cardinium* and *Arsenophonus*. The detection was performed using bacterial species-specific 16S rRNA gene-based PCR (Polymerase Chain Reaction). The mitochondrial gene 12S rRNA was used as positive control for amplification (Table S1). The amplification was performed in 25 µL reaction mixtures containing 2.5 µL KAPA Taq buffer 10×, 0.25 µL dNTPs (25 mM), 0.25 µL of KAPA Taq, 0.5 µL of the forward primer (25 µM), 0.5 µL of the reverse primer (25 µM), 1 µL of template DNA solution and was finalized with 20 µL sterile deionized water. The PCR temperature profile was 95 °C for 5 min followed by 35 cycles of 95 °C for 30 s, 30 s at the optimum annealing temperature for each pair of primers, 1 min at 72 °C and a final extension step of 72 °C for 5 min. PCR products were electrophoresed on a 1.5% agarose gel in order to examine the presence and size of the amplified fragments. The primer sequences used in this study along with the product size and annealing temperature are summarized in Table S1. The PCR-positive products were purified using polyethylene glycol (20% PEG, 2.5 M NaCl) [47] and resuspended in 15 μL water. Amplicon sequencing was performed using Sanger method.

### 2.3. PCR Amplification of V3-V4 Region, PCR Indexing and Illumina Sequencing

For the amplicon sequencing analysis, five individuals from each gender were randomly selected from the four natural populations of Hessian fly samples. The hypervariable V3-V4 region of the bacterial 16S rRNA gene was amplified from the total number of 40 adults using MiSeq universal primers 341F and 805R (Table S1). The first PCR reaction was performed and purified as previously described. In order to include the indexes as well as the Illumina adaptors, a second PCR were performed in 50 µl volume containing 5 µL KAPA Taq buffer 10×, 0.4 µL dNTPs (25 mM), 0.2 µL of KAPA Taq, 5 µL of the forward index primer (10 µM), 5 µL of the reverse index primer (10 µM), 2 µL of the cleaned PCR product diluted up to 10 ng.µL^−1^ and 32.4 µL sterile deionized water. The temperature profile used PCR was: 95 °C for 3 min followed by 8 cycles of 95 °C for 30 s, 30 s at 55 °C, 30 s at 72 °C and a final extension step of 72 °C for 3 min. The resulting amplicons were cleaned using the NucleoMag NGS (Next Generation Sequencing) Clean-up and Size Selection kit (Macherey-Nagel, Düren, Germany) following the manufacturer’s instructions. Indexed amplicons from all samples examined were mixed in equimolar ratio (8 nM) and sequencing was performed by Macrogen using a 2 × 300 bp pair-end kit on a MiSeq platform. The datasets have been deposited to NCBI under BioProject PRJNA613835.

### 2.4. Bioinformatic Analysis of Amplicon Sequencing Data

After sequencing, bioinformatic analysis was performed using USEARCH v.11 [48] and QIIME2 distribution 2019.1 [49]. Briefly, paired-end reads were assembled, trimmed by length using the usearch -fastq_mergepairs option, then, the quality of assembled sequences was improved using -fastq_filter, followed by finding unique read sequences and abundances using -fastx_uniques option. Sequences were clustered into operational taxonomic units (OTUs) with -cluster_otus command based on 97% OTU clustering using UPARSE algorithm [50]. Cross-talk errors were identified and filtered with -uncross option based on UNCROSS2 algorithm [51]. Taxonomy was assigned with Qiime2 based on BLAST+ algorithm [52] against SILVA 128 release database [53].

Richness, Simpson, Shannon and Evenness indices of alpha diversity, which reflect the diversity of individual samples were calculated based on “diversity” function from “vegan” R package and plotted using “ggplot” function from “ggplot2” package. Pair wise ANOVA was used to identify significant differences of alpha diversity indices between the different locations. Beta diversity was analyzed to evaluate the similarity of bacterial communities from different locations using Generalized UniFrac distance [54] and visualized via non-metric multidimensional scaling (NMDS) plot. A permutational multivariate analysis of variance using distance matrices was calculated using “adonis” function from “vegan” R package to determine significance differences between the separated groups. Linear discriminant analysis (LDA) effect size (LEfSe) method was applied on OTUs table to identify the discriminant taxa characterizing the four different regions using the galaxy web application (http://huttenhower.sph.harvard.edu/galaxy/) [55]. A *p*-value < 0.05 was considered indicative of statistical significance.

### 2.5. Phylogenetic Analysis

The *Wolbachia* phylogenetic analyses were carried out based on the partial 16S rRNA gene sequences obtained from the *Wolbachia*-infected samples and the sequences representing the *Wolbachia* related OTUs obtained by HTS sequencing. First, the sequences were aligned using MUSCLE [56], with the default algorithm parameters, as implemented in MEGA 7.0 software [57] and manually adjusted. Smart Model Selection [58] was used to estimate the best model of nucleotide acid evolution for constructing phylogenies of our data based on Akaike Information Criterion [59]. For the alignment of *Wolbachia* sequences obtained by Sanger sequencing, the model (GTR+G+I) was selected, while the model (TN+G+I) was selected for the alignment of *Wolbachia* related sequence obtained by the HTS sequencing. The robustness was assessed with 1000 bootstrap replicates. Maximum-Likelihood trees were constructed using MEGA 7.0 software [57]. All 16S rRNA gene sequences generated in this study have been deposited in the GenBank database under accession numbers MT231732-MT231744 and MT229221.

## 3. Results

### 3.1. Reproductive Infection Status Assessed by PCR Screening of Natural and Laboratory Hessian Fly Populations

#### 3.1.1. Reproduction Infection Prevalence in Natural and Laboratory Hessian Fly Populations

PCR screening methods were used to assay the presence of four reproductive symbionts: *Wolbachia*, *Spiroplasma*, *Cardinium* and *Arsenophonus*, in a laboratory colony and four natural Hessian fly populations. The screening results revealed that these flies were infected only by *Wolbachia* with around 3% in natural populations, and 10% in the laboratory population. Noteworthy that, the *Wolbachia* percentage infection in natural populations did not reveal an even distribution among the different locations. Only, Hessian flies from Doukkala (5 out of 27 screened individuals) were determined to be infected with *Wolbachia*. In total, 13 flies were found infected: 3 females and 2 males out of 27 individuals from Doukkala population and 8 males out of 80 individuals from the laboratory population (Table 2, Figure S1). Whereas, none of the Hessian fly populations examined were infected with *Spiroplasma*, *Cardinium* and *Arsenophonus*.

#### 3.1.2. Phylogenetic Analysis of *Wolbachia* Sequences Obtained by Sanger Sequencing

**Figure 1:**
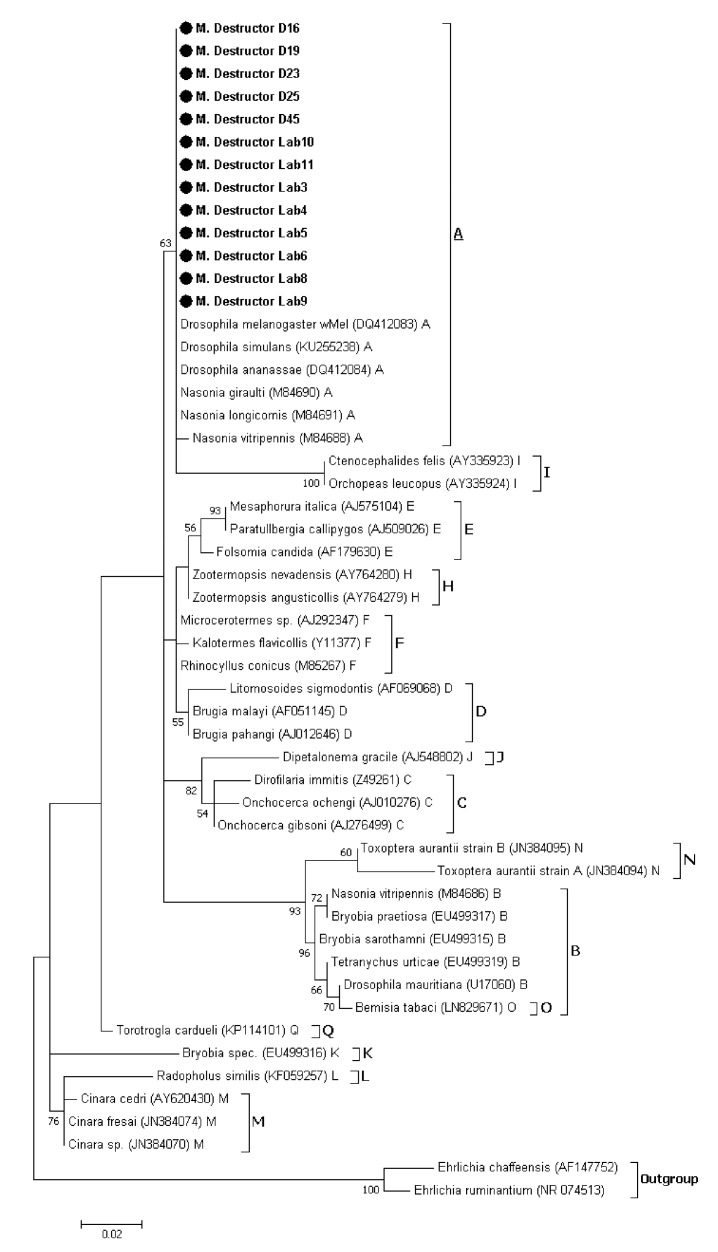
Maximum likelihood phylogenetic tree based on *Wolbachia* 16S rRNA sequences (257 bp indel-free alignment): The thirteen *Wolbachia* sequences present in Hessian fly positive samples are indicated in bold letters (D: samples from Doukkala region, Lab: samples from the laboratory colony) along with the other sequences represent the known supergroups from A to Q (except supergroup P). *Wolbachia* sequences are characterized by the names of their host species and their GenBank accession number. The number in each node represent bootstrap proportions based on 1000 replication (only values > 50% are indicated).

After removing manually the low-quality bases, 393 bp remained in thirteen *Wolbachia* sequences. The *Wolbachia* phylogenetic analysis was carried out on the thirteen *Wolbachia*-infected samples based on the partial 16S rRNA gene sequences. The results revealed that the *Wolbachia* strains infecting both natural and laboratory Hessian fly populations belonged to supergroup A (Figure 1). Interestingly, the *Wolbachia* strain sequences detected in the Hessian fly populations match perfectly with all the reference sequences of supergroup A except for *Nasonia vitripennis*.

### 3.2. 16S rRNA Gene Amplicon Sequencing Reveals the Presence of Wolbachia in Natural Populations of Hessian Fly and Dynamics Indicate an Origin Effect

The bacterial community composition and diversity of 40 natural Hessian fly samples from Chaouia, Doukkala, Fes and Safi regions were investigated using Illumina high throughput sequencing of 16S rRNA gene amplicons. A total of 1,203,664 qualified paired-end reads with an average count per samples of 30,091 reads were obtained after sequencing and quality filtering. On the basis of a 97% species similarity, 101 operational taxonomic units (OTUs) classified in six phyla, 10 classes and 79 genera were obtained across all the samples (Table S2).

#### 3.2.1. Bacterial Diversity within Hessian Flies’ Natural Populations

**Figure 2:**
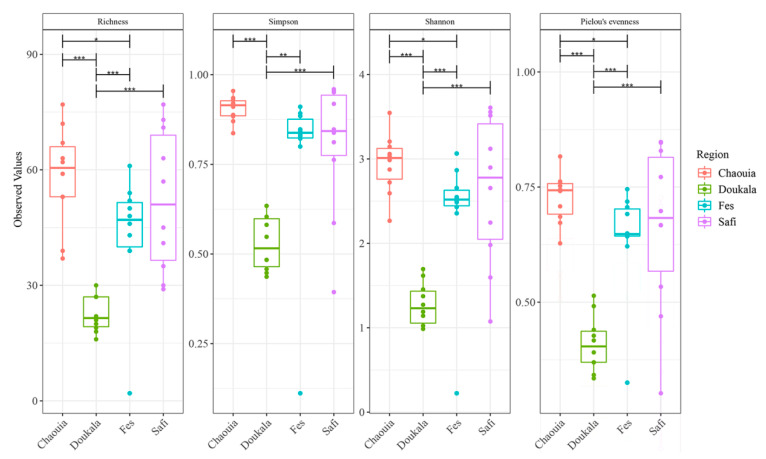
Species richness and diversity indices with significance differences of Hessian fly samples collected from Chaouia, Doukkala, Fes and Safi regions. Boxes represent inter-quartile range (IQR), the line within the boxes is the median, and the dots represent samples. * *p* < 0.05, ** *p* < 0.01 and *** *p* < 0.001.

The four examined natural populations of Hessian flies exhibited different species richness and diversity indices based on the number and relative abundance of OTUs, Simpson and Shannon indices (Figure 2). In detail, the most bacterial species rich samples were those derived from Chaouia, Fes, and Safi, while samples from Doukkala exhibited statistically lower species richness and diversity than the samples from the other regions (pairwise ANOVA: *p* < 0.001). Additionally, samples from Chaouia region exhibited statistically higher diversity, based on the Shannon index, compared to samples from Fes region (pairwise ANOVA: *p* < 0.05).

#### 3.2.2. Bacterial Composition of Hessian Flies’ Natural Populations

**Figure 3:**
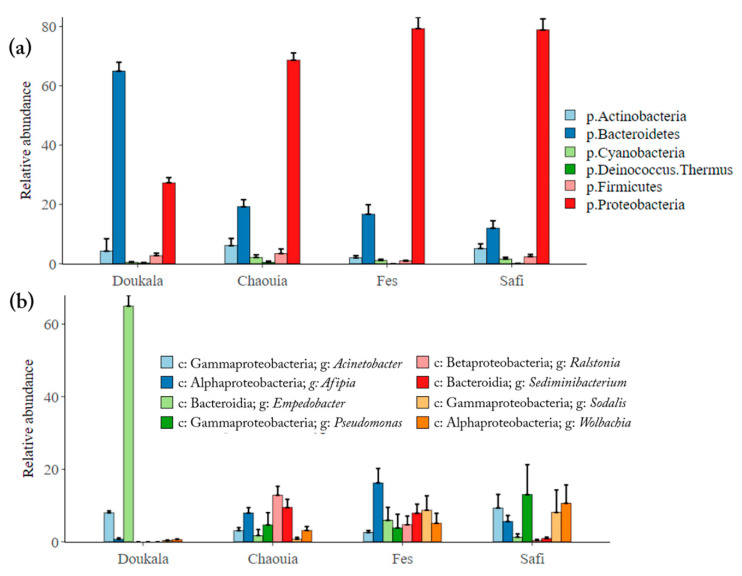
Composition of Hessian flies’ microbiota at the phylum (**a**) and genus (**b**) levels. ‘p’ corresponds to phylum, ‘c’ to class and ‘g’ to genus.

In total, most of the bacteria were identified as Proteobacteria (63.5 ± 2.9%) and Bacteroidetes (28 ± 2.7%), in addition to Actinobacteria, Cyanobacteria, Deinococcus Thermus and Firmicutes (less than 5%) (Table S2). In more detail, the Hessian flies collected from Chaouia, Fes and Safi regions shared a similar phylum distribution dominated by Proteobacteria (68.6 ± 2.4%, 79.2 ± 3.8% and 78.8 ± 3.7% respectively), Bacteroidetes (19.2 ± 2.3%, 16.6 ± 3.2% and 11.9 ± 2.4%, respectively), Actinobacteria (6.1 ± 2.4%, 1.9 ± 0.7% and 5.1 ± 1.6%, respectively) and Firmicutes (3.4 ± 1.5%, 0.9 ± 0.2% and 2.3 ± 0.8% respectively) (Figure 3a). By contrast, the Hessian flies from Doukkala region had a high relative abundance of Bacteroidetes (64.9 ± 2.9%) followed by Proteobacteria (27.2 ± 1.7%), Actinobacteria (4.2 ± 4.1%) and Firmicutes (2.7 ± 0.8%). At the class level, the most abundant taxa in the Hessian flies from Safi, Fes and Chaouia were Gammaproteobacteria, Alphaproteobacteria and Betaproteobacteria respectively. However, in Doukkala region, the Hessian flies’ microbiota was dominated mainly by Bacteroidia (Figure 3b). These results were confirmed by the LEfSe analysis and LDA scores (Figure S2A), where similar bacterial distribution patterns have been observed in the natural Hessian flies collected, suggesting that the flies’ microbiota was affected significantly by their native regions. In total, 47 OTUs were identified with LDA scores > 4.0 and *p* < 0.05 (Figure S2B). Eleven, 16, 8 and 12 OTUs were identified in Hessian flies from Doukkala, Chaouia, Fes and Safi respectively. LEfSe indicated that the most discriminant OTUs in Hessian flies were Bacteroidetes (*p* < 0.001, Kruskal-Wallis test) in Doukkala, Actinobacteria in Chaouia region, Betaproteobacteria in Fes and Gammaproteobacteria in Safi (*p* < 0.01, Kruskal Wallis test). Different frequencies of genera were observed between the collection regions. The most frequent genus in the Hessian flies was *Empedobacter* which exhibited a higher relative abundance (64.9 ± 2.9%) in Doukkala, *Ralstonia* (12.8 ± 2.4%) in Chaouia, *Afipia* (16.2 ± 3.9%) in Fes, and *Pseudomonas* (13 ± 8.2%) in Safi (Figure 3b). Interestingly, the genus *Wolbachia* was detected across all regions with a higher relative abundance in Hessian flies derived from Safi (10.6 ± 5.0%) and Fes (5.0 ± 2.7%) compared to those derived from Chaouia (3.1 ± 1.0%) and Doukkala (0.6 ± 0.1%) regions. Additionally, HTS results confirmed the absence of *Arsenophonus*, *Cardinium* and *Spiroplasma* genera in Hessian flies’ microbiota.

#### 3.2.3. Phylogenetic Analysis of *Wolbachia* Related Sequence Obtained by HTS

**Figure 4:**
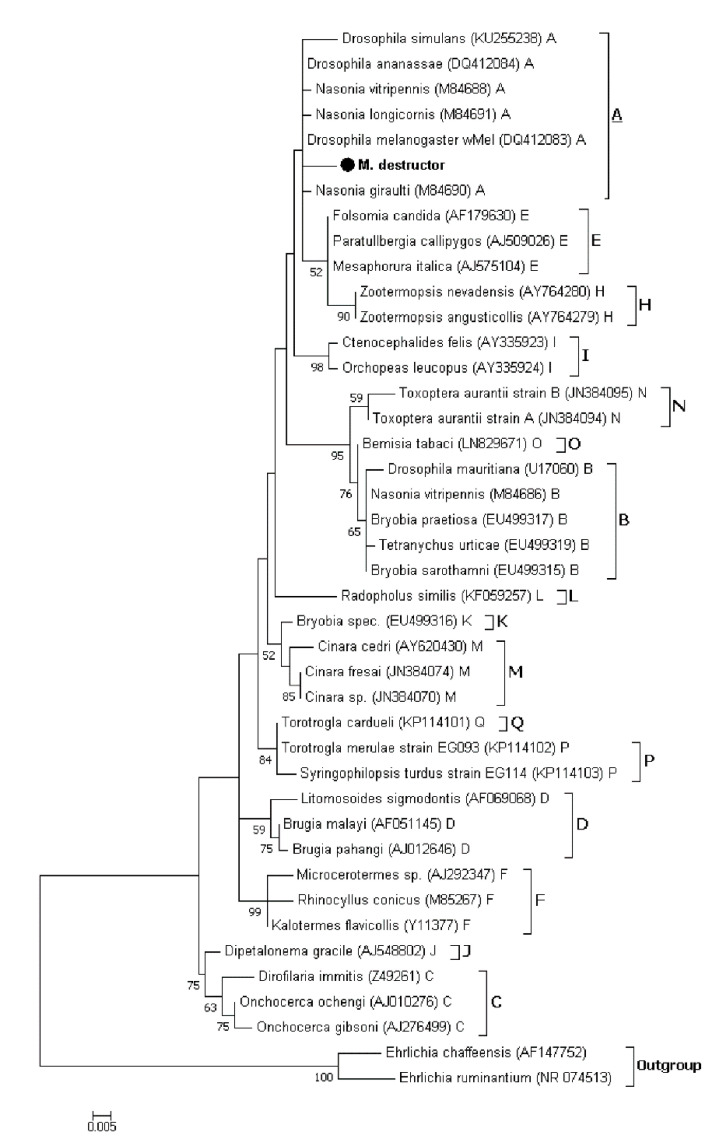
Maximum likelihood phylogenetic tree based on 16S rRNA gene of *Wolbachia* related OTU (443 bp full size alignment): The *Wolbachia* related sequence obtained from Hessian fly positive samples is indicated in bold letters along with the other sequences represent the known supergroups from A to Q. *Wolbachia* sequences are characterized by the names of their host species and their GenBank accession number. The number in each node represent bootstrap proportions based on 1000 replication (only values > 50% are indicated).

The *Wolbachia* infections status was assessed from the normalized libraries, using the conventional clustering of sequences into OTUs (97% sequence identity). *Wolbachia* related reads clustered into one OTU only, with differences in prevalence across the four studied regions. Among ten individuals per region, eight individuals were infected in Safi and Fes, nine individuals in Chaouia and the lower prevalence was detected in Doukkala with six individuals. The phylogenetic analyses based on the 16S rRNA gene placed the sequence representing the *Wolbachia* related OTU (443 bp) within the supergroup A sequences (Figure 4). These results indicate that the Hessian flies derived from different regions most likely carried the same *Wolbachia* strain.

#### 3.2.4. Bacterial Diversity between Hessian Flies’ Natural Populations

**Figure 5:**
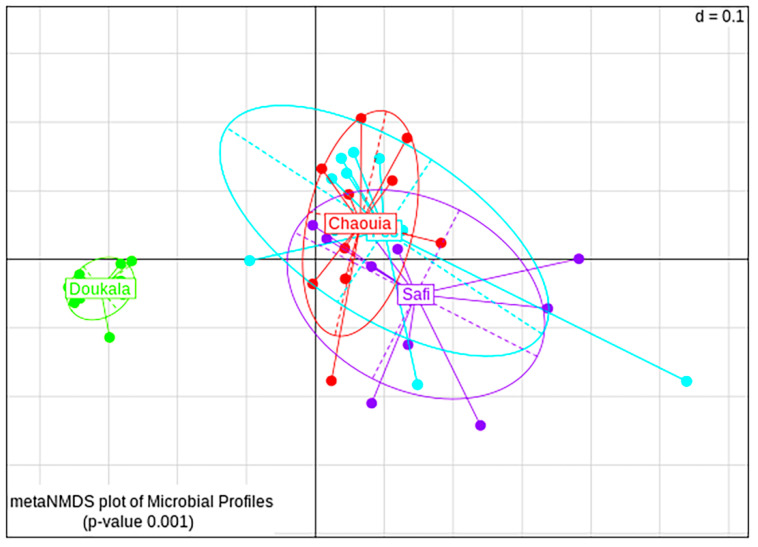
Non-metric multidimensional scaling (NMDS) plot of bacterial communities for Hessian flies samples collected from Chaouia (red), Doukkala (green), Fes (cyan) and Safi (purple) (*p* < 0.001). ‘d’ indicates dissimilarity scale of the grid (d = 0.1 mean that the distance between two grid lines represent approximately 10% dissimilarity between the samples).

Based on beta-diversity analysis, the bacterial communities seemed to be affected statistically by the origin of the insects. The NMDS plots based on generalized UniFrac distance showed that samples derived from Chaouia, Fes and Safi overlapped, whereas samples from Doukkala formed a different cluster (PERMANOVA, *p* < 0.001, Figure 5). The pairwise comparison showed that the samples from the four regions were significantly separated (PERMANOVA, *p* < 0.05, Figure S3). The distant separation of the samples derived from the region of Doukkala resulted from the significantly high abundance of Bacteroidetes, which decreased the overall diversity of the bacterial community in this region. In contrast, no differences were observed between the bacterial communities of males and females (PERMANOVA, *p* = 0.217, Figure S4).

## 4. Discussion

In the present study, we investigated the natural and laboratory populations of Hessian flies for the presence of maternally transmitted symbionts, as well as we identified the bacterial communities and diversity present in the natural Hessian fly adults collected from four different wheat producing areas in Morocco using HTS sequencing of V3-V4 region of the 16S rRNA gene.

PCR with primers specific to the studied symbionts revealed that all the screened Hessian flies collected from Moroccan fields and a laboratory population maintained in ICARDA did not harbor any *Spiroplasma*, *Arsenophonus* or *Cardinium* infections. However, five out of 28 flies from Doukkala region and eight males out of 80 flies from the laboratory colony harbor *Wolbachia*. *Wolbachia* infections were also detected in HTS DNA sequence datasets from 31 out of 40 individuals of the four studied natural populations. The level of detection was significantly higher in two individuals from Safi (42.84% and 38.13%) and one individual from Fes (28.96%). However, the *Wolbachia* related reads represents less than 11% in the other individuals (Table S3). The difference of prevalence between the two approaches may be related to the low *Wolbachia* infection density and the increased sensitivity of HTS compared to the standard PCR screening [60,61]. It should be noted that *Wolbachia* had never been detected in natural populations of the Hessian fly [44,45,62]. To our knowledge, among the members of gall midges family, *Wolbachia* was reported only in two species, Asian rice gall midge (*Orseolia oryzae*) [63] and pine needle gall midge (*Thecodiplosis japonensis*) [64]. However, wide range of agricultural pests known to harbor one or multiple strains of *Wolbachia* including Aphids [20,46,65,66], fruit flies members of Tephritidae family [67,68,69,70,71,72] as well as members of Drosophilidae family [73,74,75,76]. Currently, there are 16 identified major supergroups of *Wolbachia* strains named from A to Q with exception of supergroup G which has been considered as combination of A and B supergroups [46,77,78,79,80,81]. The characterization of *Wolbachia* strains is primarily based on the 16S rRNA gene, multi locus sequence typing systems (MLST) using five conserved genes as molecular markers (*gatB*, *coxA*, *hcpA*, *ftsZ* and *fbpA*) as well as the *Wolbachia* surface protein (*wsp*) gene [82,83]. Our attempts to characterize *Wolbachia* strains detected in Hessian fly laboratory and natural populations using MLST and *wsp* genes, were constrained by amplification issues possibly due to the low infection density which complicate the detection and strain characterization of *Wolbachia* infections [84,85,86,87,88]. Thus, our phylogenetic analyses were based only on 16S rRNA sequences amplified using *Wolbachia* specific primers and the *Wolbachia* related sequence obtained using HTS. All the *Wolbachia* sequences obtained in this study appeared to be most homologous to the strains belonging to supergroup A (Figure 1 and Figure 4), suggesting that the origin of Hessian fly samples did not conduct to *Wolbachia* strain divergence. It has been previously observed that the density of *Wolbachia* in a host affects the level of CI that occurs [89]. Therefore, the low infection rate observed in our study suggests that *Wolbachia* most likely does not result in reproductive incompatibility between Hessian flies. Moreover, PCR detection of *Wolbachia* in both males and females, may suggest that no female-biased sex ratio effects should be found in response to infection. Although *Wolbachia* are usually transmitted vertically, the possibility that the infected population from Doukkala could be the result of horizontal transmission cannot be excluded since this mode of transmission is common among and within insect species [90,91] and could be mediated through host plants [92] or parasitoids [93]. Meanwhile, our finding sheds a new light on the ability of Hessian flies to carry a *Wolbachia*-infection, which may lead us to produce a stable *Wolbachia*-infected line using the transinfection technique. This technique aims to transfer symbiont strains to new hosts within the same species or between different species. It has already been applied for *Wolbachia* in *Aedes albopictus* [94,95], *Anopheles stephensi* [96], *Aedes polynesiensis* [97], *Aedes aegypti* [98,99], *Ceratitis capitata* [100], and *Bactrocera oleae* [101].

In addition to *Wolbachia* infection status, we assessed whether geographical origin affect the bacterial composition of Hessian flies. Based on HTS sequencing, we identified several distinct OTUs from four different Hessian fly populations (Table S2 and Figure S5). Our results revealed that the microbiota of adult Hessian flies was significantly influenced by their native regions. Samples from Chaouia, Fes and Safi exhibited a higher number of OTUs and higher species richness indicating that these samples contained more diverse microbiota compared to the samples from Doukkala region. Members of Proteobacteria were the predominant bacterial taxa in samples derived from Chaouia, Fes and Safi regions, while samples from Doukkala were dominated mainly by Bacteroidetes due to the abnormally high relative abundance of *Empedobacter* taxon, and Proteobacteria as a second most abundant phylum. The presence of Proteobacteria in all samples could be due to the fact that maggots recruit Proteobacteria from the wheat hosts during the feeding stages of Hessian flies (1st and 2nd instar) and these are subsequently transferred to adults across developmental stages [102,103,104,105]. At the genus level, an earlier study using culture-independent and standard sequencing approaches, identified *Ochrobactrum* member of Alphaproteobacteria as the dominant taxa in Hessian fly adults, followed by *Alcaligenes* member of Betaproteobacteria, *Arthrobacter* and *Microbacterium* members of Actinobacteria and *Sphingobacterium* member of Bacteroidia [44]. However, in our study, diverse bacterial genera were detected in Hessian fly across the different regions. The most abundant taxa were *Ralstonia* member of Betaproteobacteria in Chaouia, *Pseudomonas* member of Gammaproteobacteria in Safi, *Afipia* a member of Alphaproteobacteria in Fes and *Empedobacter* a member of Bacteroidetes, in Doukkala. Low abundance of the previously mentioned genera [44] (<5%) were identified as well in Hessian flies from Chaouia, Fes and Safi. The differences of the bacterial composition in Hessian flies could be influenced by the wheat cultivars used in the fields. Indeed, the effects of wheat cultivars on the Hessian fly larvae and adults’ microbiota need to be elucidated. Additional to the host plant, other factors could also contribute to shaping the bacterial composition including climatic conditions, soil microorganisms and infection with symbionts like *Wolbachia* [106,107,108,109,110]. Further works on the Hessian flies and their environment are therefore required to understand the origin and the role of different symbiont associated to Hessian fly populations.

## 5. Conclusions

The geographical location was found to affect significantly the bacterial composition in Hessian fly adults. Higher number of OTUs dominated by members of Proteobacteria were observed in the flies derived from Chaouia, Fes and Safi. However, those derived from Doukkala region were abnormally dominated by *Empedobacter*, a member of the Bacteroidetes/Chlorobi group, which may be due to the local environments and/or the wheat cultivar used. To the best of our knowledge, this study documents the first cases of *Wolbachia* infection in Hessian fly populations. Phylogenetic analyses based on 16S rRNA gene revealed the presence of only supergroup A *Wolbachia* strain in all the infected flies. Since the *Wolbachia* infection occurs in low titer, reproductive incompatibility may not be induced in the studied populations. However, the presence of this infection may give a new insight into the use of *Wolbachia* for the fight against Hessian fly populations. This could be tested using an experimental transfer of a *Wolbachia* strain known to induce a high level of cytoplasmic incompatibility into Hessian flies for the suppression of natural populations.

## Figures and Tables

**Table 1:** Number of collected Hessian fly adults from different locations.

Region	Location	Coordinates			Number of Insects	
		Altitude	Latitude	Longitude	Female	Male
Safi	Jamaat Shaim	173	32.24076	−8.46976	32	27
Fes	Fes	584	34.01436	−5.34543	23	30
Doukkala	Khemis Zemamra	162	32.614952	−8.664802	19	8
Chaouia	Sidi El Aidi	247	33.07341	−7.37935	16	9
-	Laboratory colony	-	-	-	40	40

**Table 2:** Prevalence of bacterial endosymbionts screened in natural and laboratory populations of Hessian fly.

Population	Gender	Sample Size	*Wolbachia*	*Spiroplasma*	*Cardinium*	*Arsenophonus*
Doukkala	Female	19	+ (3)	−	−	−
	Male	8	+ (2)	−	−	−
Safi	Female	32	−	−	−	−
	Male	27	−	−	−	−
Fes	Female	23	−	−	−	−
	Male	30	−	−	−	−
Chaouia	Female	16	−	−	−	−
	Male	9	−	−	−	−
Laboratory	Female	40	−	−	−	−
	Male	40	+ (8)			
Total		244	+ (13)	−	−	−

+ infected samples (with number of infected individuals per population), − uninfected samples.

## Supplementary Materials

The following are available online at https://www.mdpi.com/2075-4450/11/6/340/s1, Table S1: Primer pairs used in PCR amplifications, Table S2: Relative abundance (%) of different bacteria detected in Hessian fly Samples derived from Safi, Fes, Chaouia and Doukkala regions, Table S3: Hessian flies positive for *Wolbachia* screened using PCR and the number of *Wolbachia* related reads and their frequencies per samples, Figure S1: Detection of *Wolbachia* in Hessian flies using PCR and electrophoretic separation on 1.5% agarose gel., Figure S2: LEfSe results of Hessian flies’ microbiota, Figure S3: Pairwise non-metric multidimensional scaling (NMDS) plot of bacterial communities for Hessian fly samples collected from Chaouia (red), Doukkala (green), Fes (cyan) and Safi (purple), Figure S4: Non-metric multidimensional scaling (NMDS) plot of bacterial communities for males (cyan) and females (red) of Hessian fly samples, Figure S5: Heat map showing relative distribution and relative abundance of OTUs detected in Hessian fly samples at genus level.
