# Supplementary material for: Detection of Wolbachia Infections in Natural and Laboratory Populations of the Moroccan Hessian Fly, Mayetiola destructor (Say)
Source: Insects. 2020 Jun 2;11(6):340. doi: 10.3390/insects11060340 (PMC7349215; doi:10.3390/insects11060340)
Supplement: Supplementary file 1 [file insects-11-00340-s001.zip › Supplementary Files-proof/Supplementary Materials Table S1 FigureS1í¬S5.docx]

**Supplementary Materials**

**Table S1.** Primer pairs used in PCR amplifications

| **Genus** | **Primer** | **Sequence (5’-3’)** | **Tm °C** | **Product size** | **Reference** |
| --- | --- | --- | --- | --- | --- |
| *Wolbachia* | WspecF | YATACCTATTCGAAGGGATAG | 55 °C | 438 bp | [1] |
|  | WspecR | AGCTTCGAGTGAAACCAATTC |  |  |  |
| *Spiroplasma* | 63F | GCCTAATACATGCAAGTCGAACGG | 60 °C | 450 bp | [2] |
|  | TKSS | TAGCCGTGGCTTTCTGGTAA |  |  |  |
| *Cardinium* | CloF1 | GGAACCTTACCTGGGCTAGAATGTATT | 56 °C | 466 bp | [3] |
|  | CloR1 | GCCACTGTCTTCAAGCTCTACCAAC |  |  |  |
| *Arsenophonus* | ArsF | GGGTTGTAAAGTACTTTCAGTCGT | 54 °C | 600 bp | [4] |
|  | ArsR3 | CCTYTATCTCTAAAGGMTTCGCTGGATG |  |  |  |
| 12S rRNA | 12SCFR | GAGAGTGACGGGCGATATGT | 54 °C | 377 bp | [5] |
|  | 12SCRR | AAACCAGGATTAGATACCCTATTAT |  |  |  |
| 16S rRNA  V3-V4 region | U341F | CCTACGGGRSGCAGCAG | 53 °C | 464 bp | [6] |
|  | 805R | GACTACCAGGGTATCTAAT |  |  |  |

**Reference**

1. Werren, J.H.; Windsor, D.M. Wolbachia infection frequencies in insects: evidence of a global equilibrium? *Proc Biol Sci* **2000**, *267*, 1277–1285.

2. Fukatsu, T.; Nikoh, N. Endosymbiotic Microbiota of the Bamboo Pseudococcid Antonina crawii (Insecta, Homoptera). *Applied and Environmental Microbiology* **2000**, *66*, 643–650.

3. Gotoh, T.; Noda, H.; Ito, S. *Cardinium* symbionts cause cytoplasmic incompatibility in spider mites. *Heredity* **2007**, *98*, 13–20.

4. Duron, O.; Bouchon, D.; Boutin, S.; Bellamy, L.; Zhou, L.; Engelstädter, J.; Hurst, G.D. The diversity of reproductive parasites among arthropods: Wolbachiado not walk alone. *BMC Biology* **2008**, *6*, 27.

5. Hanner, R.; Fugate, M. Branchiopod Phylogenetic Reconstruction from 12s Rdna Sequence Data. *J Crustacean Biol* **1997**, *17*, 174–183.

6. Klindworth, A.; Pruesse, E.; Schweer, T.; Peplies, J.; Quast, C.; Horn, M.; Glöckner, F.O. Evaluation of general 16S ribosomal RNA gene PCR primers for classical and next-generation sequencing-based diversity studies. *Nucleic Acids Research* **2013**, *41*, e1–e1.

**
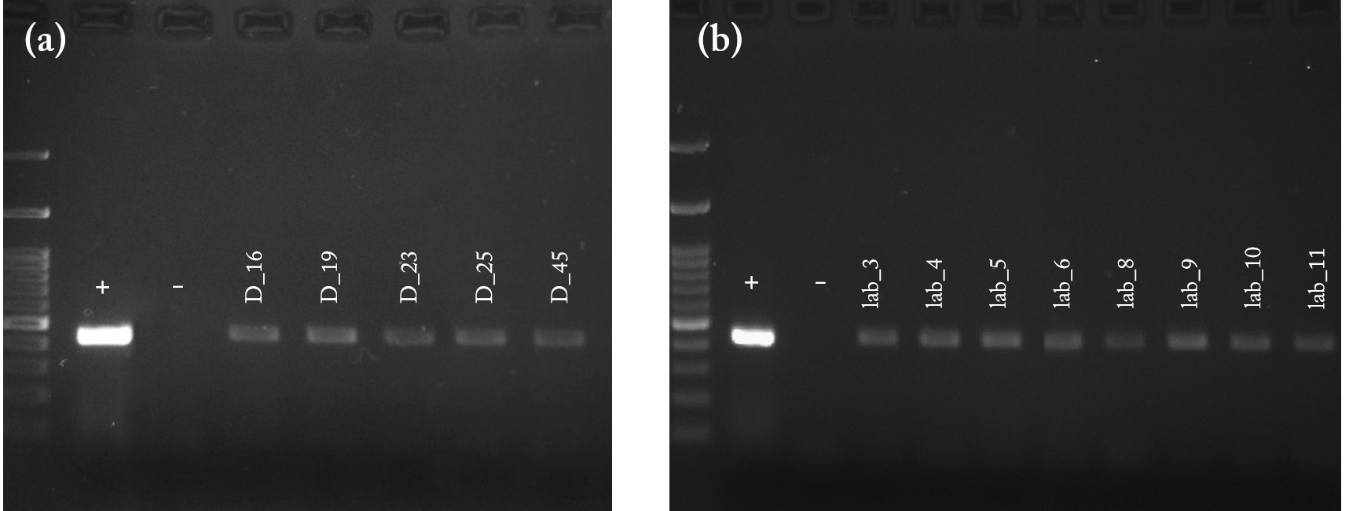
Figure S1.** Detection of Wolbachia in Hessian flies using PCR and electrophoretic separation on 1.5% agarose gel. **(a)** Doukala population. **(b)** Laboratory population. + Positive control, − Negative control.


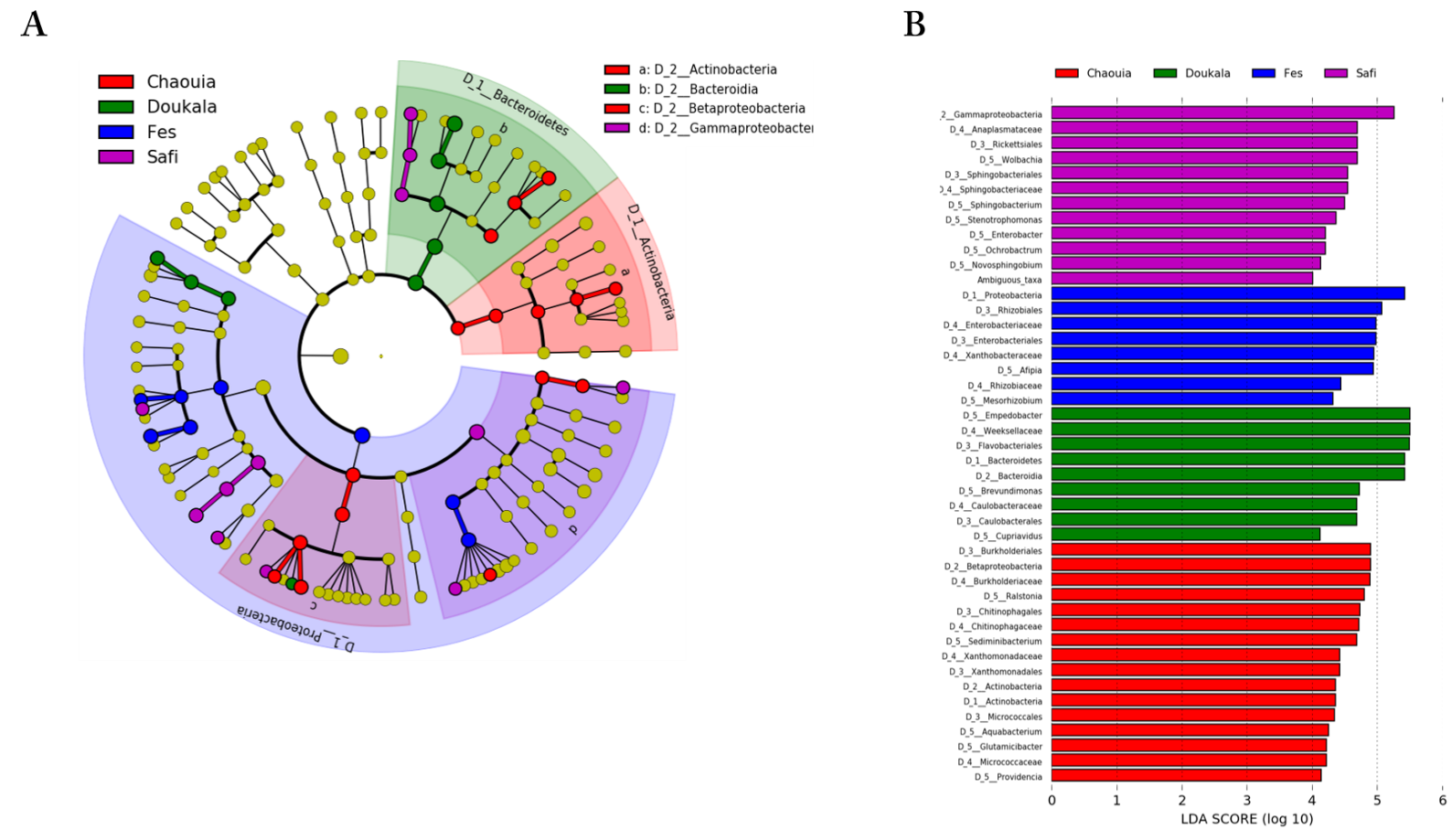
**Figure S2.** LEfSe results of Hessian flies’ microbiota. **(A)** Cladogram representation of the differentially abundant phylum and class. The size of each node is proportional to the taxon abundance. **(B)** Log10 of LDA scores for the most discriminant bacterial taxa identified in Doukala, Chaouia, Fes and Safi regions (LDA score > 4.0, *p* < 0.05).

**
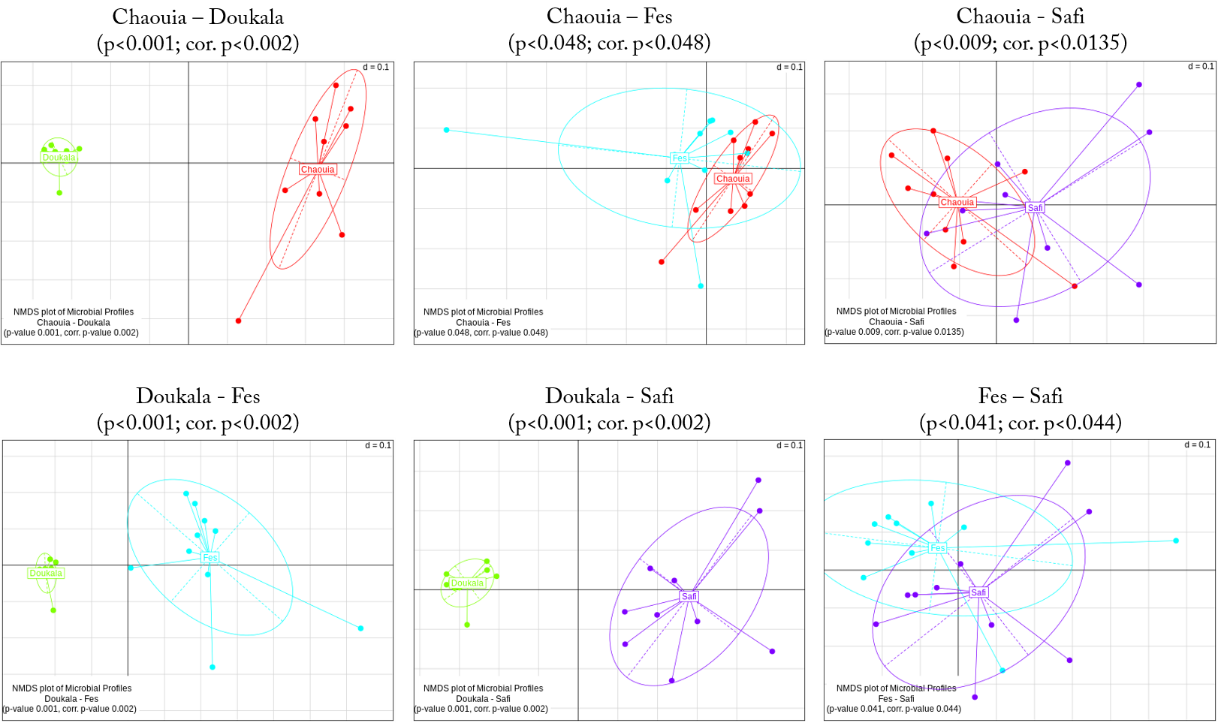
Figure S3.** Pairwise non-metric multidimensional scaling (NMDS) plot of bacterial communities for Hessian fly samples collected from Chaouia (red), Doukala (green), Fes (cyan) and Safi (purple) (*p* < 0.001). ‘d’ indicates dissimilarity scale of the grid (d = 0.1 mean that the distance between two grid lines represent approximately 10% dissimilarity between the samples). ‘cor. *p*’ indicates the pairwise test significance values obtained after correction for multiple testing using the Benjamini–Hochberg method.


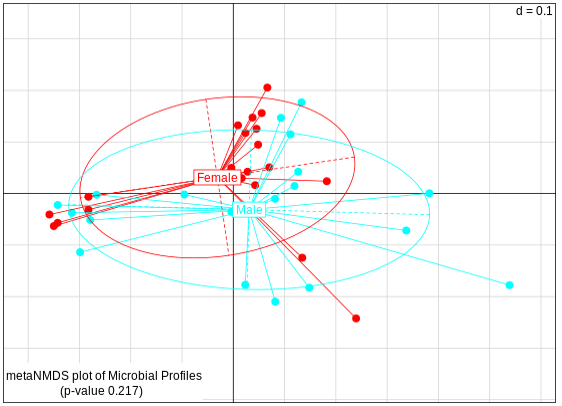
**Figure S4.** Non-metric multidimensional scaling (NMDS) plot of bacterial communities for males (cyan) and females (red) of Hessian fly samples (*p* = 0.217). ‘d’ indicates dissimilarity scale of the grid (d = 0.1 mean that the distance between two grid lines represent approximately 10% dissimilarity between the samples).


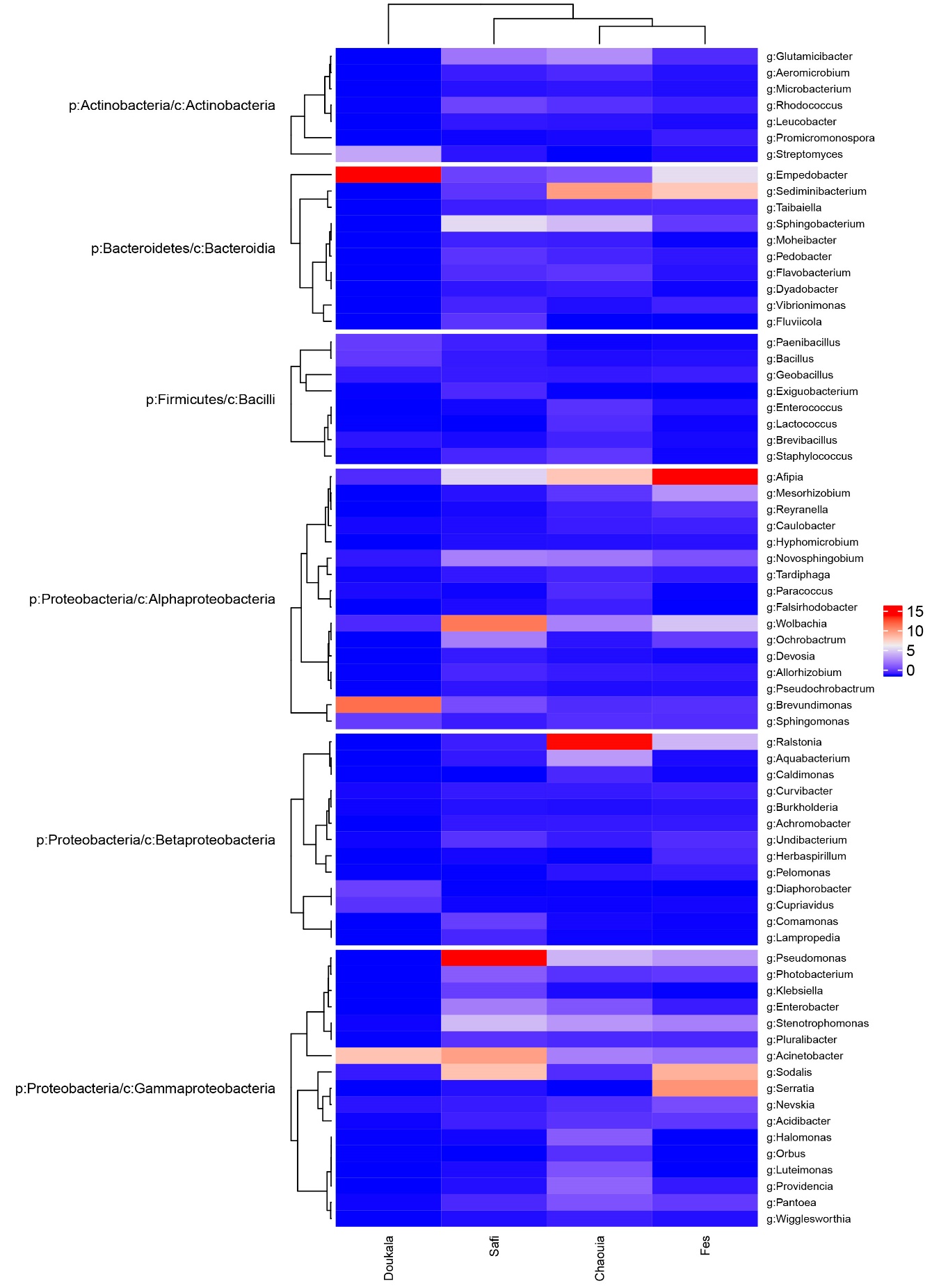
**Figure S5.** Heat map showing relative distribution and relative abundance of OTUs detected in Hessian fly samples at genus level.
